# Likelihood ratios of quantitative laboratory results in medical diagnosis: The application of Bézier curves in ROC analysis

**DOI:** 10.1371/journal.pone.0192420

**Published:** 2018-02-22

**Authors:** Walter Fierz

**Affiliations:** labormedizinisches zentrum Dr Risch, Vaduz, Liechtenstein; Weill Cornell Medical College in Qatar, QATAR

## Abstract

Receiver operating characteristic (ROC) analysis is widely used to describe the discriminatory power of a diagnostic test to differentiate between populations having or not having a specific disease, using a dichotomous threshold. In this way, positive and negative likelihood ratios (LR+ and LR-) can be calculated to be used in Bayes’ way of estimating disease probabilities. Similarly, LRs can be calculated for certain ranges of test results. However, since many diagnostic tests are of quantitative nature, it would be desirable to estimate LRs for each quantitative result. These LRs are equal to the slope of the tangent to the ROC curve at the corresponding point. Since the exact distribution of test results in diseased and non-diseased people is often not known, the calculation of such LRs for quantitative test results is not straightforward. Here, a simple distribution-independent method is described to reach this goal using Bézier curves that are defined by tangents to a curve. The use of such a method would help in standardizing quantitative test results, which are not always comparable between different test providers, by reporting them as LRs for a specific diagnosis, in addition to, or instead of, quantities such as mg/L or nmol/L, or even indices or units.

## Introduction

Medical diagnostics is an information processing endeavor based on probabilities. Two types of medical information can be distinguished, patient-specific and knowledge-based [1]. Diagnostics is about connecting these two types of information [2]. The production of patient-specific information is the main objective of the clinical laboratory. Laboratory tests can significantly contribute to this effort by modifying the probabilities of a diagnosis. Many modern laboratory techniques provide quantitative test results and it would be important to know how much a particular test result would increase or decrease the odds for a specific diagnosis. For example, how much does a D-dimer result of 1000 μg/L, double the recommended cut-off, increase or decrease the clinical suspicion of thrombosis. The answer to that question lies in applying Bayes’ theorem [3–5]: pretest odds multiplied by the likelihood ratio (LR) of the laboratory test result give the posttest odds (S1 Appendix). LRs are defined by the ratio of the probability of the test result in the population carrying the disease versus the probability in the non-diseased population.

$$LR=\frac{sensitivity}{1-specificity}=\frac{Se}{1-Sp}$$

The question is how the LR of a measured quantitative test result can be determined.

The analysis of Receiver Operating Characteristics (ROC) of diagnostic tests is common in establishing the merits of laboratory tests and in determination of cut-off values [6]. ROC curves are defined by the relation between the true positive rates (TP, sensitivity) and the false positive rates (FP, 1-specificity) for various cut-offs in dichotomous test interpretation. The area under the curve (AUC) serves to compare the diagnostic value of different tests [7]. The higher the AUC is, the better the diagnostic value of the test. However, ROC curves contain much more information in that for a specific quantitative test result, the LR of this result is equal to the slope of the tangent to the ROC curve at the point on the ROC curve corresponding to the measured test result [8]. Unfortunately, publications of ROC curves of diagnostic tests or test information given by the test producers do not contain this information. Particularly, the quantitative test results underlying the ROC curves are usually not published in detail, so it is impossible to calculate the LR of a particular quantitative test result. In addition, no simple method is currently available to reach this goal.

Knowing the distribution of a test parameter in the diseased and non-diseased population would allow calculation of the slopes of the tangents, i.e. the LRs, with statistical methods. However, for many tests these distributions are not exactly known and differ for various test parameters. Consequently, a distribution-free estimation of the slopes on the ROC curves is required. Here it is demonstrated that the approximation of the empirical points of a ROC curve by a cubic Bézier curve directly leads to the desired slopes of the tangents and thereby to the LRs of a measured quantitative test result. To illustrate the method, some examples of ROC data are used where raw data of the ROC curves have been published, which is rarely the case in the literature.

## Methods

Pierre Bézier (1910–1999) was a French engineer who developed a method of producing computer-driven curves to be used in the design of automobiles at Renault, which came to be known as Bézier curves. The algorithm to calculate these curves was developed by mathematician Paul de Casteljau at Citroën. The mathematical basis for Bézier curves are the Bernstein polynomials (for review see [9]). Bernstein polynomials of degree n are defined by

$$B_{i,n}\left(t\right)=\left(\begin{array}{c} n\\ i \end{array}\right)t^{i}{\left(1-t\right)}^{n-i},\text{with}\;\text{t}\;\text{ranging}\;\text{from}\;0\;\text{to}\;1.$$ (1)

For the purpose here, we make use of cubic Bézier curves defined by

$$\text{B}\left(\text{t}\right)={\left(1-\text{t}\right)}^{3}{\text{P}}_{0}+3\;\text{t}{\left(1-\text{t}\right)}^{2}{\text{P}}_{1}+3\;{\text{t}}^{2}\left(1-\text{t}\right){\text{P}}_{2}+{\text{t}}^{3}{\text{P}}_{3}$$ (2)

**Fig 1:**
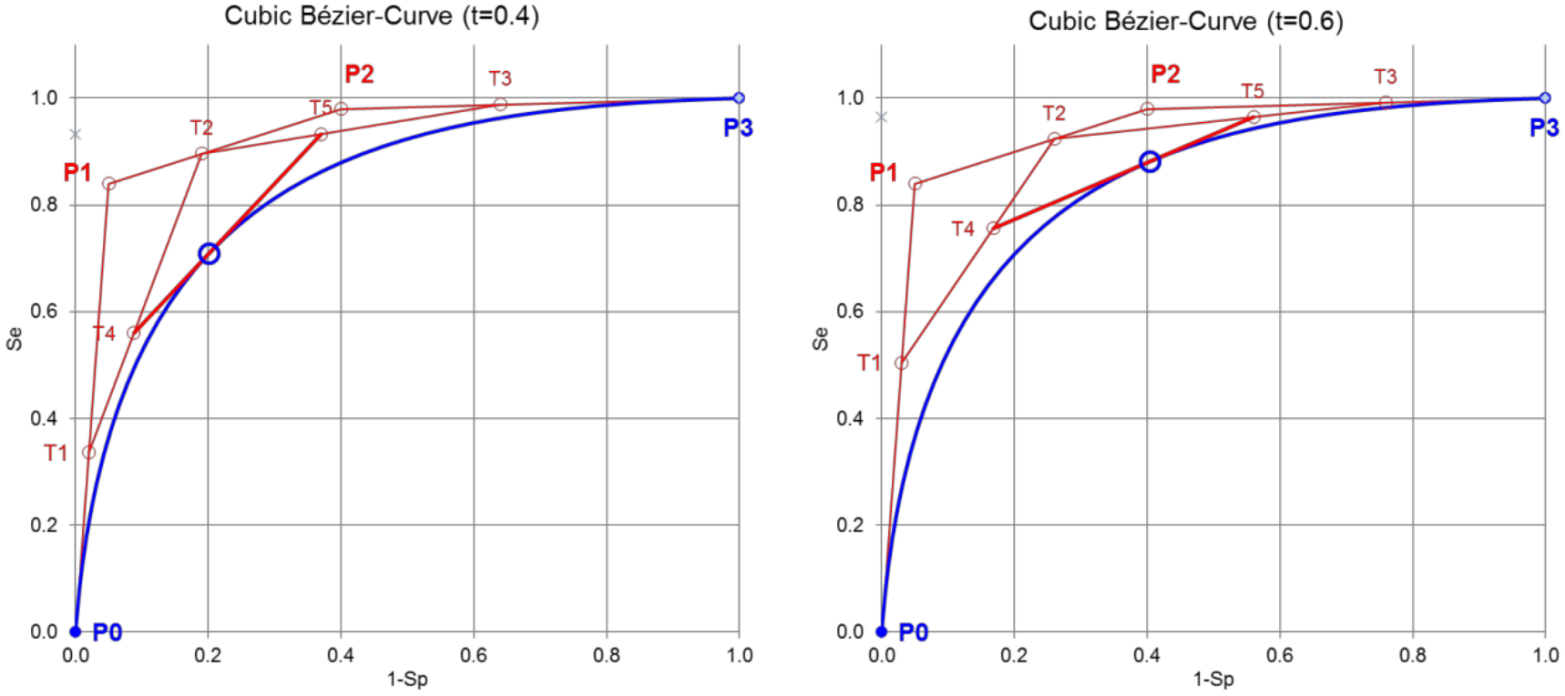
Principle of constructing cubic Bézier curves. First, the lines between the control points P0, P1, P2, and P3 are divided by the ratio t leading to T1, T2, and T3. Second, the lines between T1, T2, and T3 are again divided by the ratio t leading to T4, and T5. Third, the line between T4, and T5 is again divided by the ratio t leading to B(t) on the Bézier curve. The line between T4, and T5 is the tangent to B(t).

The cubic Bézier curve is determined by the four control points P_0_, P_1_, P_2_, and P_3_ (Fig 1). The variable, relative position of the points T_1_, T_2_, T,_3_ T_4_, and T_5_ between the control points P_(0,1,2,3)_ is equal to the ratio t. The Bézier curve is given by the tangents defined by T_4_ and T_5_ for all t from 0 to 1.

Given a particular empirical ROC curve, a Bézier curve can be fitted to the points of the ROC curve by adjusting the control points P_(0,1,2,3)_ with the following least square methods.

### Step 1

First, the Bernstein polynomials are rewritten in the following form for the x and y coordinates:

$$B_{x}\left(t\right)=a_{x}*t^{3}+b_{x}*t^{2}+c_{x}*t+d_{x^{,}}\;\text{and}\;B_{y}\left(t\right)=a_{y}*t^{3}+b_{y}*t^{2}+c_{y}*t+d_{y}$$ (3)

The x values of the ROC points (1- Sp) and the y values (Se) are fitted by a least square method with the above polynomials. This can be done e.g. with the regression analysis (RGP) function in a Microsoft Excel table. The variable t of the Bernstein polynomials (Eq 3) has to be introduced and is defined here by t_xy_ = (x+y)/2 of the empirical ROC points. When (1-Sp) reaches zero with Se > 0 and/or Se reaches its maximum with (1-Sp) < 1, the range of t has to be proportionally adjusted to the range from 0 to 1 with the following transformation:

$$\text{t}=\left({\text{t}}_{xy}-\text{min}\left({\text{t}}_{xy}\right)\right)/\left(\text{max}\left({\text{t}}_{xy}\right)-\text{min}\left({\text{t}}_{xy}\right)\right)$$ (4)

### Step 2

Second, having established the coefficients a, b, c, and d, of the Bernstein polynomials (3) the coordinates of the control points, P_(0,1,2,3)_ are calculated using the following relations for both, x and y coordinates (see S2 Appendix):

$${\text{P}}_{0}=\text{d},{\text{p}}_{1}=\frac{\text{c}}{3}+\text{d},{\text{P}}_{2}=\frac{\text{b}+2*\text{c}+3*\text{d}}{3}\;\text{and}\;{\text{P}}_{3}=\text{a}+\text{b}+\text{c}+\text{d}$$ (5)

With P_(0,1,2,3)_ being established in this way, the slopes of the tangents, i.e. the LR(t), can be calculated for all t (see S3 Appendix).

### Step 3

Third, the relation between the quantitative test results and their position on the Bézier curve and thereby the LR(t)s has to be established, which of course depends on the test parameter. This can be done in three ways. Most directly, the LRs of the individual empirical points on the ROC curve, calculated in step 2, and their relation to the quantitative test result can be generalized by fitting a relation function using least square approximation. More indirectly, a λ value based on LR, i.e. λ = 1/(1+LR) can be fitted to the quantitative test results. This λ can also be used to calculate λ-weighted Youden indices [10, 11] (see Eq 6). Third, the t values used to construct the Bézier curve can be fitted to the quantitative test results. In either way, preferring the method that gives the best fit, the diagnostic LR can be calculated from all quantitative test results.

## Results

The three steps described above are exemplified by their application to a simple example of a ROC curve with raw data available from the literature [12].

**Fig 2:**
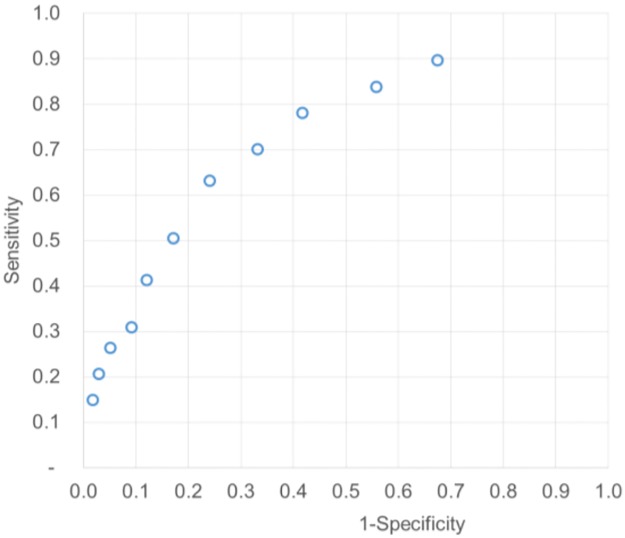
HbA1c test as a tool in the diagnosis of gestational diabetes mellitus. ROC curve of the original data [12].

The starting data are given in Table 1 and Fig 2.

**Table 1:** HbA1c test as a tool in the diagnosis of gestational diabetes mellitus. t values are calculated according to step 1 in methods.

HbA1c [mmol/mol]	FP (1-Sp)	TP (Se)	t_xy_ (FP+TP)/2	t
31	0.67	0.90	0.79	1.00
32	0.56	0.84	0.70	0.88
33	0.42	0.78	0.60	0.74
34	0.33	0.70	0.52	0.62
36	0.24	0.63	0.44	0.50
37	0.17	0.51	0.34	0.36
38	0.12	0.41	0.27	0.26
39	0.09	0.31	0.20	0.17
40	0.05	0.26	0.16	0.11
41	0.03	0.21	0.12	0.05
42	0.02	0.15	0.08	0.00

### Step 1 and 2

**Fig 3:**
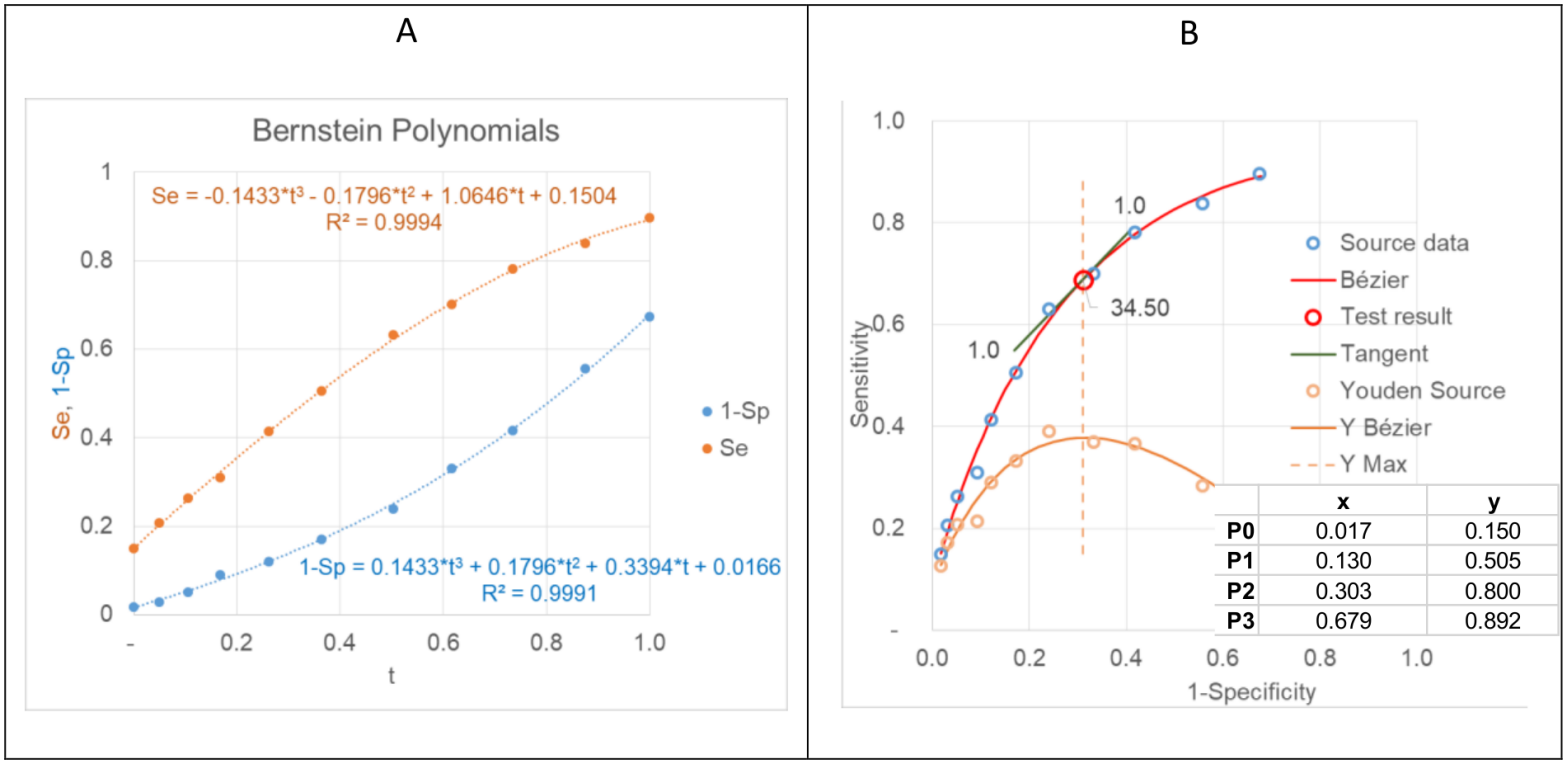
Bernstein polynomials (A) for Se and 1-Sp for calculating the control points P_0,1,2,3_ of the Bézier curve (B). Youden indices (Y) with their maximum (Ymax) are indicated. The slope of the tangent to the ROC curve at Ymax equals 1.

Cubic Bernstein polynomials are fitted to the data points by establishing cubic polynomials for Se(t) and 1-Sp(t) (Fig 3A). The x and y coordinates of the control points P_(0,1,2,3)_ for the Bézier curve are calculated from the coefficients of the cubic polynomials according to (5). The Bézier curve is constructed as described in Fig 1 and shown in Fig 3B.

The HbA1c value where the LR = 1 i.e. where the slope of the tangent to the curve equals 1 is 34.5 mmol/mol Hb. This corresponds to the point where the Youden index (Y = Se+Sp-1) [10] reaches its maximum, i.e. where the cut-off is optimal for maximizing the number of correctly classified individuals (Fig 3B).

### Step 3

**Fig 4:**
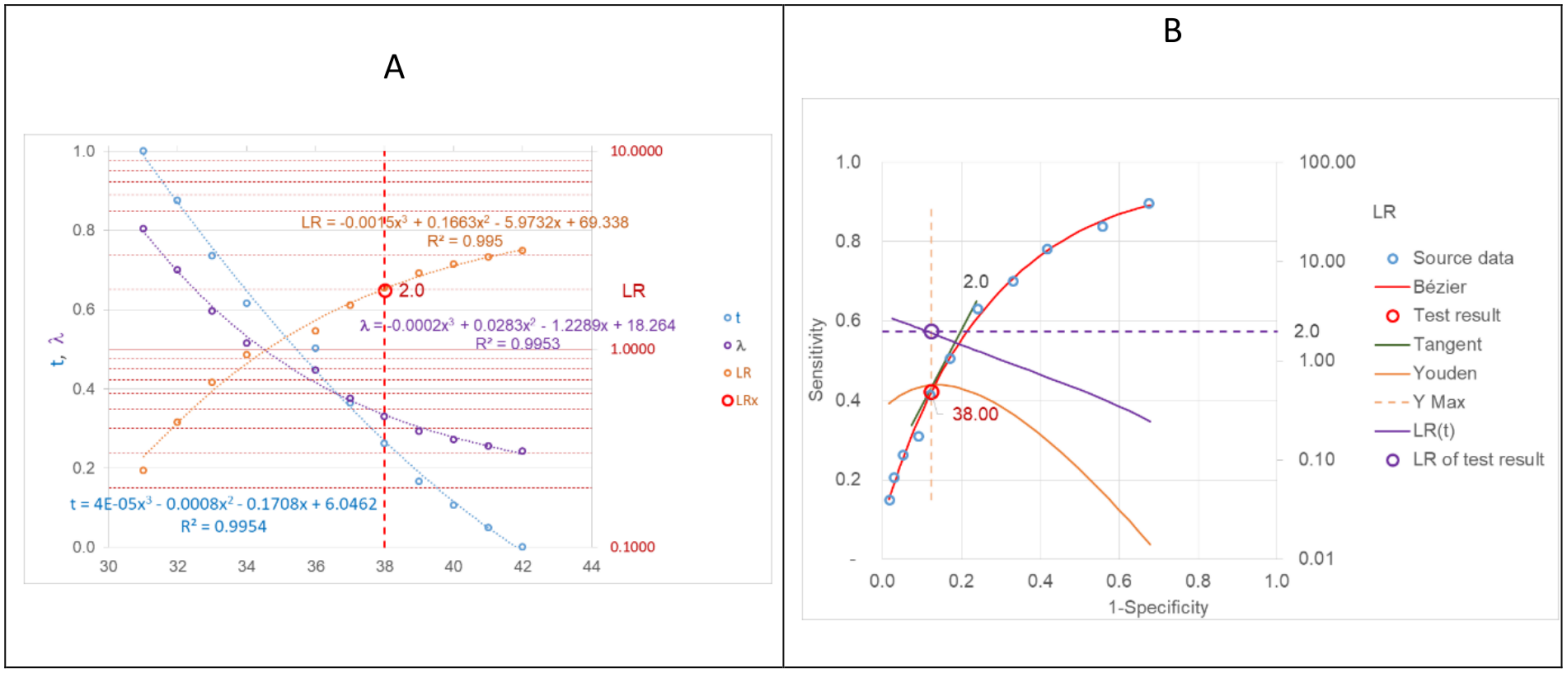
Calculating LRs from test results with three different methods. Curve fitting with cubic polynomials of test results of known data points to LRs (1), to λ or to t (3) for calculating the desired LR of a given test result, directly or indirectly from λ or t (A). At Hba1c = 38 mmol/mol, e.g., the LR reaches 2 (B). λ-weighted Youden indices, Y(λ), with their maximum, Y(λ)max, are indicated. The slope of the tangent to the ROC curve at Y(λ)max equals LR.

For all known data points the LR(t) are calculated using the formulas in S3 Appendix, and a general relation between Hba1c values and corresponding LRs is established by a least square approximation (Fig 4). In this way, for each quantitative test result the LR can be calculated, independent of the parameter distribution and independent of any cut-offs. At Hba1c = 38 mmol/mol, e.g., the LR reaches 2.

A remarkable observation is that when λ-weighted Youden indices [10, 11] are used, as defined by

$$\text{Y}\left(\lambda \right)=2*\left(\lambda *\text{Se}+\left(1-\lambda \right)*\text{Sp}\right)-1,\text{with}\lambda =\frac{1}{1+\text{LR}}$$ (6)

the point on the ROC curve with the corresponding LR = (1 –λ)/λ always is at the maximum of the λ-weighted Youden index. The case of the “optimal” cut-off where the non-weighted Youden index is at its maximum, as shown in Fig 3B, is then just a special case of the more general λ-weighted Youden index, when λ = 0.5, i.e. LR = 1 and Y = Se + Sp − 1.

**Fig 5:**
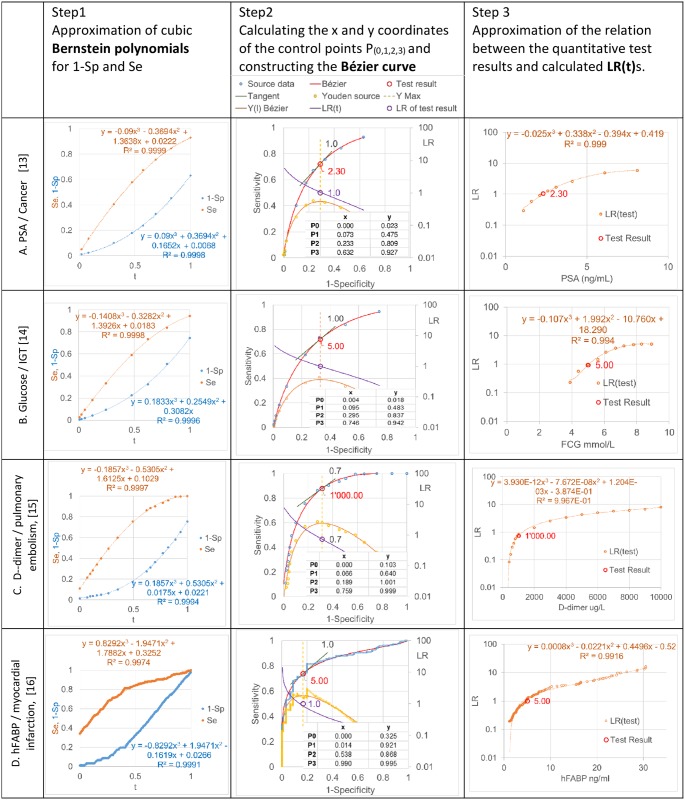
Examples of Bézier curve approximation and likelihood ratio calculation.

In the following (Fig 5), some examples from the literatur are calculated by using the above procedure. The source of data are published ROC curves with detailed laboratory test results:

A Prostate-specific antigen in men with an initial PSA level of 3.0 ng/ml or lower (data from [13])
B Fasting capillary blood glucose as a screening test for impared glucose tolerance (data from [14])
C D-dimer testing for suspected pulmonary embolism in outpatients (data from [15])
D Heart-type fatty acid-binding protein in suspected acute myocardial infarction (data from [16] see supporting information S1 File)

## Discussion

In order to calculate LR values for quantitative test results, a distribution-free algorithm based on Bézier curves is proposed. The necessary calculations can easily be done, e.g. by using a Microsoft Excel tool. The advantage of the method is that it is generally applicable, independently and without knowledge of the test parameter distribution in the population. The accuracy of the method is independent on the clinical situation but only depends on the accuracy of the empirical ROC points. The clinical examples chosen here to demonstrate the method are selected on ground of detailed ROC data availability in the literature.

Bézier curves are mathematically well defined and widely used in computer graphics. Here, we make use of cubic Bézier curves defined by Bernstein polynomials of degree 3. Approximation of the cubic Bernstein polynomials B(t) to empirical points on the ROC curve is done by a least square method for the B_x_(t) and B_y_(t) coordinates separately, t being a variable between 0 and 1. The crucial advantage of this procedure is that Bézier curves are constructed by tangents to the curve, whose slopes immediately provide the LR of a specific point on the curve. It remains to relate all quantitative results of a test to positions on the Bézier ROC curve and thereby to their LRs. Three methods to do so are proposed.

The example question posed in the introduction as to how much a D-dimer result of 1000 ug/L increases or decreases the clinical suspicion of thrombosis can be answered after this analysis with LR = 0.7, i.e. the result with LR being smaller than 1 lowers the initial clinical suspicion of thrombosis, although 1000 ug/L is double the cut-off of 500 ug/L. A LR = 1 is only reached with 1300 ug/L. In fact, it is a frequently observed mistake in test interpretation that results that lie closely to the point on the ROC curve where LR = 1 are wrongly interpreted or overestimated in their diagnostic significance.

The merit of using LRs in addition to or even instead of quantities like mg/L or nmol/L as test results lies in the comparability of tests that are using different methods and that are produced by different test suppliers, which is an unsolved problem of standardization in laboratory medicine, particularly in immune serology [17]. Of course, LRs are always related to a specific diagnosis and ROC curves must be established for each diagnosis separately. This is reasonable, since it is good clinical practice to base the choice of a laboratory test and its corresponding ROC curve on a tentative diagnosis. However, this requirement requests that the test producers cannot just calibrate their products by comparing them to other products, but have to do clinical studies with the test.

In conclusion, ROC curves of diagnostic tests that are approximated by Bézier curves provide likelihood ratios for quantitative test results, independent on test methods. These likelihood ratios allow to estimate the probabilities of diagnosis based on pretest probabilities according to Bayes’ theorem. Such inferences based on quantitative test results have otherwise not been possible so far.

## Supporting information

S1 Appendix Bayes’ theorem. (DOCX)

S2 Appendix Bernstein polynomials. (DOCX)

S3 Appendix Likelihood ratio. (DOCX)

S1 File Novel biomarkers hFABP, copeptin, GP-BB and MRP8/14 in the very early diagnosis of acute myocardial infarction. (PDF)

## References

[1] Fierz W. Challenge of personalized health care: to what extent is medicine already individualized and what are the future trends? Med Sci Monit. 2004; 15114285

[2] Fierz W. Information management driven by diagnostic patient data: right information for the right patient. Expert Rev Mol Diagn. 2002; 2: 355–360. doi: 10.1586/14737159.2.4.355 12138500 10.1586/14737159.2.4.355

[3] Hall GH. The clinical application of Bayes' theorem. Lancet 1967; 2: 555–557. 4166903 10.1016/s0140-6736(67)90514-4

[4] van der Helm HJ, Hische EAH. Application of Bayes’ Theorem to Results of Quantitative Clinical Chemical Determinations. Clin Chem. 1979; 25: 985–988. 445835

[5] Malakoff D. Bayes offers a 'new' way to make sense of numbers. Science 1999; 286: 1460 10610542 10.1126/science.286.5444.1460

[6] Zweig MH, Campbell G. Receiver-operating characteristics (ROC) plots—a fundamental evaluation tool in clinical medicine. Clin Chem. 1993; 39: 561–577. 8472349

[7] Yu J, Yang L, Vexler A, Hutson AD. Easy and accurate variance estimation of the nonparametric estimator of the partial area under the ROC curve and its application. Stat Med. 2016; 35: 2251–2282. doi: 10.1002/sim.6863 26790540 10.1002/sim.6863

[8] Choi BC. Slopes of a receiver operating characteristic curve and likelihood ratios for a diagnostic test. Am J Epidemiol. 1998; 148: 1127–1132. 9850136 10.1093/oxfordjournals.aje.a009592

[9] Casselman B. From Bézier to Bernstein. Feature Column from American Mathematical Society 2008 http://www.ams.org/samplings/feature-column/fcarc-bezier

[10] Youden WJ. Index for rating diagnostic tests. *Cancer*. 1950; 3: 32–35. doi: 10.1002/1097-0142(1950)3:1<32::AID-CNCR2820030106>3.0.CO;2-3 15405679 10.1002/1097-0142(1950)3:1<32::aid-cncr2820030106>3.0.co;2-3

[11] Gail MH, & Green SB. Generalization of One-Sided 2-Sample Kolmogorov-Smirnov Statistic for Evaluating Diagnostic Tests. Biometrics. 1976; 32, 561–570. doi: 10.2307/2529745 963171

[12] Renz PB, Cavagnolli G, Weinert LS, Silveiro SP, Camargo JL. HbA1c test as a tool in the diagnosis of gestational diabetes mellitus. PLoS One. 2015; 10: e0135989 doi: 10.1371/journal.pone.0135989 26292213 10.1371/journal.pone.0135989 PMC4546239

[13] Thompson IM, Ankerst DP, Chi C, Lucia MS, Goodman PJ, Crowley JJ, et al Operating characteristics of prostate-specific antigen in men with an initial PSA level of 3.0 ng/ml or lower. Jama 2005; 294, 66–70. doi: 10.1001/jama.294.1.66 15998892 10.1001/jama.294.1.66

[14] Bortheiry AL, Malerbi DA, Franco LJ. The ROC curve in the evaluation of fasting capillary blood glucose as a screening test for diabetes and IGT. Diabetes Care. 1994; 17: 1269–1272. doi: 10.2337/diacare.17.11.1269 7821166 10.2337/diacare.17.11.1269

[15] Perrier A, Desmarais S, Goehring C, de Moerloose P, Motrabia A, Unger PF, et al D-Dimer Testing for Suspected Pulmonary Embolism in Outpatients. Am J Respir Crit Care Med. 1997; 156: 492–496 doi: 10.1164/ajrccm.156.2.9702032 9279229 10.1164/ajrccm.156.2.9702032

[16] Schoenenberger AW, Stallone F, Walz B, Bergner M, Twerenbold R, Reichlin T, et al Incremental value of heart-type fatty acid-binding protein in suspected acute myocardial infarction early after symptom onset. *Eur Hear J Acute Cardiovasc Care*. 2016; 5: 185–192. doi: 10.1177/2048872615571256 25681485 10.1177/2048872615571256

[17] Fierz W. Basic problems of serological laboratory diagnosis. Methods Mol Med. 1998; 13: 443–471. doi: 10.1385/0-89603-485-2:443 21390860 10.1385/0-89603-485-2:443

