# Supplementary material for: Likelihood ratios of quantitative laboratory results in medical diagnosis: The application of Bézier curves in ROC analysis
Source: PLoS One. 2018 Feb 22;13(2):e0192420. doi: 10.1371/journal.pone.0192420 (PMC5823376; doi:10.1371/journal.pone.0192420)
Supplement: S1 Appendix — (DOCX) [file pone.0192420.s001.docx]

# **S1 Appendix. Bayes’ theorem**

The classical form of Bayes’ theorem is:

$P\left( H | D \right)=P\left( D | H \right)*P(H)/P(D)$ (1)
where the probability (*P*) of the hypothesis (*H*), given the data (*D*), is equal to the probability of the data, given that the hypothesis is correct, multiplied by the probability of the hypothesis before obtaining the data divided by the average probability of the data [5].

Similarly

$P\left( \overline{H} | D \right)=P\left( D | \overline{H} \right)*P\left( \overline{H} \right)/P(D)$ (2)

By dividing equation (1) by equation (2), we get

$\frac{P(\left( H | D \right)}{P\left( \overline{H} | D \right)}=\frac{P\left( D | H \right)*P(H)/P(D)}{P\left( D | \overline{H} \right)*P\left( \overline{H} \right)/P(D)}$ (3)

Since $\frac{P(\left( H | D \right)}{P\left( \overline{H} | D \right)}$ are the post-test odds, and $\frac{P(H)}{p(\overline{H)}}$ the pretest odds,
Bayes’ equation finally becomes

$Posttest odds=\frac{P\left( D | H \right)}{P\left( D | \overline{H} \right)}*pretest odds$ 
$\frac{P\left( D | H \right)}{P\left( D | \overline{H} \right)}$ by definition is the likelihood ratio, therefore

$Posttest odds=LR*pretest odds$ (4)
