# Supplementary material for: Likelihood ratios of quantitative laboratory results in medical diagnosis: The application of Bézier curves in ROC analysis
Source: PLoS One. 2018 Feb 22;13(2):e0192420. doi: 10.1371/journal.pone.0192420 (PMC5823376; doi:10.1371/journal.pone.0192420)
Supplement: S2 Appendix — (DOCX) [file pone.0192420.s002.docx]

# **S2 Appendix. Bernstein polynomials**

Cubic Bernstein polynomials are of the form

$B\left( t \right)=\left( 1-t \right)^{3}*P_{0}+3*t*\left( 1-t \right)^{2}*P_{1}+3*t^{2}*\left( 1-t \right)P_{2}+t^{3}*P_{3}$ (1)

or expanded:

$$B\left( t \right)=\left( 1-t \right)*\left( 1-2t+t^{2} \right)*P_{0}+3*t*\left( 1-2t+ t^{2} \right)*P_{1} + \left( 3t^{2}-3t^{3} \right)*P_{2}+t^{3}*P_{3}$$

$$B\left( t \right)=\left( 1-2t+t^{2} \right)*P_{0}-\left( t-2t^{2}+t^{3} \right)*P_{0}+(3t-6t^{2}+3t^{3})*P_{1}+(3t^{2}-3t^{3})*P_{2}+ t^{3}*P_{3}$$

$$B\left( t \right)=\left( 1-3t+3t^{2}-t^{3} \right)*P_{0}+(3t-6t^{2}+3t^{3})*P_{1}+(3t^{2}-3t^{3})*P_{2}+t^{3}*P_{3}$$

$$B\left( t \right)=P_{0}-3 P_{0} t+3 P_{0}t^{2}-P_{0} t^{3}+3 P_{1}t-6 P_{1}t^{2}+3 P_{1}t^{2}+3 P_{2}t^{2}-3 P_{2}t^{3}+P_{3} t^{3}$$

$$B\left( t \right)=P_{0}+3*\left( P_{1}-P_{0} \right)*t+3*\left( P_{0} -2P_{1}+P_{2} \right){*t}^{2} + \left( -P_{0}+3P_{1}-3P_{2}+P_{3} \right)*t^{3}$$

$B(t)=d+c*t+b{*t}^{2}+a*t^{3}$ (2)

with

$d=P_{0}$ => $P_{0} =d$

$c=3\left( P_{1}-P_{0} \right)$ => $P_{1}=c/3+d$

$b=3\left( P_{0}-2P_{1}+P_{2} \right)$ => $P_{2}=(b+2*c+3*d)/3$

$a=\left( -P_{0}+3P_{1}-3P_{2}+P_{3} \right)$ => $P_{3}=a+b+c+d$
