# Supplementary material for: Likelihood ratios of quantitative laboratory results in medical diagnosis: The application of Bézier curves in ROC analysis
Source: PLoS One. 2018 Feb 22;13(2):e0192420. doi: 10.1371/journal.pone.0192420 (PMC5823376; doi:10.1371/journal.pone.0192420)
Supplement: S3 Appendix — (DOCX) [file pone.0192420.s003.docx]

# **S3 Appendix. Likelihood ratio**

Calculation of LR(t)

${T1}_{x}={P0}_{x}+({P1}_{x}-{P0}_{x})*t$ $T1_{y}={P0}_{y}+({P1}_{y}-{P0}_{y})*t$

${T2}_{x}={P1}_{x}+({P2}_{x}-{P1}_{x})*t$ ${T2}_{y}={P1}_{y}+({P2}_{y}-{P1}_{y})*t$

${T3}_{x}={P2}_{x}+({P3}_{x}-{P2}_{x})*t$ ${T3}_{y}={P2}_{y}+({P3}_{y}-{P2}_{y})*t$

${T4}_{x}={T1}_{x}+({T2}_{x}-{T1}_{x})*t$ ${T4}_{y}={T1}_{y}+({T2}_{y}-{T1}_{y})*t$

${T5}_{x}={T2}_{x}+({T3}_{x}-{T2}_{x})*t$ ${T5}_{y}={T2}_{y}+({T3}_{y}-{T2}_{y})*t$

$LR(t)=({T5}_{y}-{T4}_{y}) / ({T5}_{x}-{T4}_{x})$ (1)

or

$LR(t)=\frac{{P1}_{y}*(1-2*t)-({P1}_{y}-{P2}_{y})*(2*t-3*t^{2})+{P3}_{y}*t^{2}-{P0}_{y}*{(1-t)}^{2}}{{P1}_{x}*(1-2*t)-({P1}_{x}-{P2}_{x})*(2*t-3*t^{2})+{P3}_{x}*t^{2}-{P0}_{x}*{(1-t)}^{2}}$ (2)
