# Supplementary material for: Likelihood ratios of quantitative laboratory results in medical diagnosis: The application of Bézier curves in ROC analysis
Source: PLoS One. 2018 Feb 22;13(2):e0192420. doi: 10.1371/journal.pone.0192420 (PMC5823376; doi:10.1371/journal.pone.0192420)
Supplement: S1 File — (PDF) [file pone.0192420.s004.pdf]

luzerner kantonsspital
ZENTRUM FÜR LABORMEDIZIN

Novel biomarkers hFABP, copeptin, GP-BB and MRP8/14 in the very early diagnosis of acute myocardial infarction

Walz B.* , Schönenberger A.[1], Bergner M.[2], Küest S.[2], Karajan T.[2], Elsasser S.[3], Cuculi F.[2], Petrov G.[2], Fierz, W.[3], Erne P.[2]

* Institut für Klinische Chemie und Immunologie, ZLM, Luzerner Kantonsspital, Lucerne, Switzerland
[1] Division of Geriatrics, Department of General Internal Medicine, Inselspital, University Hospital and University of Berne, Switzerland
[2] Department of Internal Medicine, Luzerner Kantonsspital, Lucerne, Swizerland
[3] Labormedizinisches Zentrum Dr. Risch, Schaan, Liechtenstein
* Correspondence : brigitte.walz@luks.ch

Introduction

Early detection of acute myocardial infarction (AMI) in the early phases still bears uncertainties. High sensitive troponins rise too late, myoglobin lacks specificity and ST-elevation may not be present and if so, it has only a 50 - 60% sensitivity for diagnosis of AMI. A patient with acute chest pain therefore often has to wait long hours until diagnosis is clear, time urgently needed for treatment. A marker that rises faster than hs-troponin and has a good sensitivity and specificity and is not dependent of other organ functions is needed.

Aim

The main goal of the study was to assess the suitability of emerging markers of AMI in a collective of patients admitted to a tertiary care hospital in a prospective study.

Methods

In search of a very early biomarker of AMI 350 consecutive patients with chest pain or other classical symptoms of AMI lasting 4 hours or less before presentation were admitted. At admission blood was drawn and the following markers were measured in addition to the classical panel: copeptin, heart-FABP (hFABP), GP-BB and MRP8/14. Three and six hours later further samples were taken. Myoglobin. hs-troponin T (hsTnT), Ck, Ck-MB, CRP, NT-proBNP, creatinin, ALAT, LDH were measured without delay on a Cobas 6000 platform. Two new markers were ELISAs (GP-BB, Dia-genics, Woburn, MA, USA, MRP8/14 Bühlmann Laboratories, Switzer-land). hFABP was measured as a rapid chromatographic immunoassay. With a subset of patients hFABP was measured qualitatively (Renesa UG, Berlin, D), all patient samples were measured quantitatively (Concile, Freiburg, D). Aliquots of the samples (serum) were frozen at -80°C and measured batchwise.

Patients were treated appropriately according to hospital standards (resuscitation, ECG, stenting, etc.). End points were: confirmed AMI, STEMI or NSTEMI. Unstable angina pectoris was not an accepted end point. Statistics were done using STATA and a model system (Fig, 3).

Results

319 Patients were enrolled in the study, some characteristics are shown in Figure 1. STEMI or NSTEMI was diagnosed in 178 patients (55.8%), patients without STEMI/NSTEMI were controls: 141 (44.2%).

Characteristics	All study patients (N = 319)	STEMI or NSTEMI (N = 178)	Controls (N = 141)	P value[1]
Age, years, mean ± SD	61.8 ± 13.4	61.7 ± 12.5	61.8 ± 14.5	0.958
Male sex, n (%)	240 (75.2%)	145 (81.5%)	95 (67.4%)	0.004
Symptoms at admission				
Thorax pain, n (%)	226 (70.9%)	139 (78.1%)	87 (61.7%)	0.001
Other symptoms, n (%)	88 (27.6%)	36 (20.2%)	52 (36.9%)	0.001
No symptoms, n (%)	5 (1.6%)	3 (1.7%)	2 (1.4%)	1.000
Electrocardiography				
ST-seg. elevation or new LBBB, n (%)	119 (37.3%)	107 (60.1%)	12 (8.5%)	<0.001
ST-seg. depression or T-wave alteration, n(%)	101 (31.7%)	61 (34.3%)	40 (28.4%)	0.260

Fig.1. Characteristics of enrolled patients. There was no statistically significant difference in age, kidney function, cardiovascular risk factors. Controls are all patients without AMI. P-value is given for AMI (STEMI or NSTEMI versus controls.

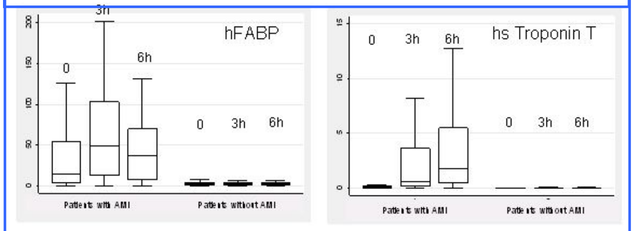

Fig. 2: hFABP and hsTnT at admission (0), three (3h) and six hours later (6h) in patients with AMI and those without. hFABP and hsTnT are significantly higher (p< 0.001) in AMI compared to controls at all times, hFABP is more pronounced at admittance.

hFABP

Fatty acid-binding proteins are a group of at least 9 small cytoplasmic proteins (13 - 14 kDa) that organise the intracellular transport of fatty acids. Because they are small, they leak readily out of severed cells, as does hFABP when cardiomyocytes are damaged.

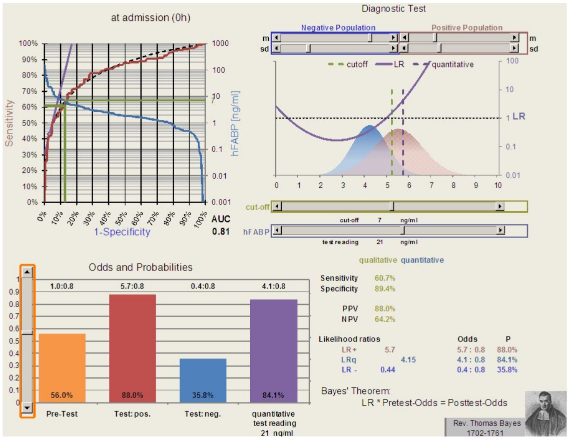

Fig.3. Interactive model of ROC-Curve for hFABP with measured Values (upper left graph, blue curve(right scale).The distribution of nevative and positive populations (upper right) and PPV, NPV below.

ROC Curves of the markers show, that hFABP and hsTnT have very similar characteristics. Sensitivity of hFABP at 7 ng/mL in the first hour after onset of pain (n=33) = 62%, specificity 83%. HsTnT had a sensitivity of 52% and a specificity of 75% at this time. The PPV of hFABP at admission (0) with a given 55.8% pretest probability is 88% (Fig. 3.), for hsTNT 78%. MRP8/14 and GP-BB had much lower sensitivity and specificity. Copeptin had better test characteristics in certain cases, statistically, it was not a suitable marker for screening for AMI in our setting.
In the first hours after onset of symptoms, hFABP had better diagnostic accuracy than hsTnT. Best accuracy was reached if the patient was hospitalized within one hour of onset of pain. In the clinically important subgroup of patients, which were not resuscitated before hospital admission, hFABP had a significantly higher diagnostic accuracy over hsTnT (Fig. 5).

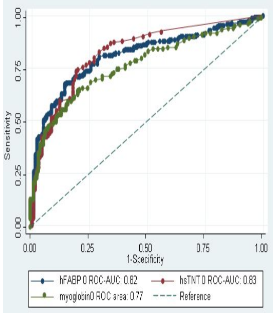

Fig. 4. ROC characteristics for hFABP, hsTNT, myoglobin at admission, all patients within 4h of onset of pain..

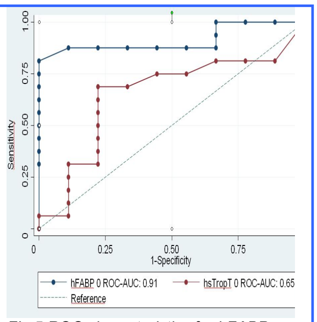

Fig.5.ROC characteristics for hFABP in a subset of patients that were not resuscitated and had chest pain for one hour or less upon admission.

Conclusions

From all tested candidates for very early diagnosis of AMI, hFABP showed the best characteristics. In our cohort, hFABP was a more sensitive marker in the very early phases after AMI than hsTnT. It does add to the diagnostic certitude up to 3 hours after onset of symptoms, afterwards, no added value to hs-TnT was found.
Potentially, the marker may be used before the patient is hospitalized, so for example in doctors offices, ambulances etc. to stratify risks so that patients can be treated without delay after admission into a hospital.
